# The Role of Artificial Intelligence in Improving Diagnosis, Management, and Outcomes of Acute Myocardial Ischemia: A Systematic Review

**DOI:** 10.7759/cureus.98865

**Published:** 2025-12-10

**Authors:** Shaima Tariq Mansoor Beig, Muazzam M Sheriff, Ammar Eid Z Alhejaili, Amani Dawod Mohammed Kamel, Sheikheldin Ibrahim Elnair, Moayad Abdulraouf Ahmed, Lina Mohammad Hatem Mawardi, Leen Abdulkareem Fida, Enas Abdulhafeez, Raydaa Hamed Jan, Hanan Yousef Ismael Tukruni, Rahaf Abdulaziz Aljahdali, Khaled Zamil Mofleh Alshahrani, Waleed Hatem Hakami, Anmar Abdulzaher Saati

**Affiliations:** 1 Medicine and Surgery, Ibn Sina National College for Medical Studies, Jeddah, SAU; 2 Microbiology and Immunology, Ibn Sina National College for Medical Studies, Jeddah, SAU

**Keywords:** acute myocardial ischemia, artificial intelligence, cardiovascular medicine, diagnosis, machine learning, prognosis

## Abstract

Artificial intelligence (AI) has emerged as a transformative force in cardiovascular medicine, particularly in the diagnosis, management, and prognostication of acute myocardial ischemia (AMI). This systematic review synthesizes current evidence on AI applications across diagnostic modalities, risk stratification, therapeutic decision-making, and outcome prediction in AMI. A total of 30 peer-reviewed studies were included, encompassing machine learning (ML), deep learning (DL), and hybrid models applied to electrocardiography (ECG), imaging, and electronic health records (EHRs). AI demonstrated superior diagnostic accuracy, enhanced triage efficiency, and improved prognostic modeling compared to conventional methods. Notably, AI-enabled ECG interpretation and coronary imaging have shown cardiologist-level performance in detecting ischemia. Risk prediction models using ML have outperformed traditional scoring systems, while AI-driven decision support tools have optimized therapeutic pathways. Despite promising results, challenges remain in clinical integration, interpretability, and generalizability. This review underscores the potential of AI to revolutionize AMI care and highlights future directions for research, validation, and ethical implementation.

## Introduction and background

Acute myocardial ischemia, a critical manifestation of coronary artery disease, is a leading contributor to global cardiovascular morbidity and mortality [1]. Prompt and accurate diagnosis, coupled with timely therapeutic intervention, is essential to mitigate myocardial damage and improve clinical outcomes [2]. However, conventional diagnostic tools, including electrocardiography (ECG), cardiac biomarkers, and imaging modalities, often exhibit limitations in sensitivity, specificity, and interpretability, particularly in atypical presentations or resource-limited settings [3].

In recent years, artificial intelligence (AI) has emerged as a powerful adjunct in cardiovascular medicine, offering data-driven insights that transcend human cognitive limitations [4]. AI encompasses a spectrum of computational techniques, including machine learning (ML), deep learning (DL), and natural language processing (NLP), which can analyze complex, high-dimensional datasets to identify patterns, predict outcomes, and support clinical decision-making [5].

The integration of AI into acute myocardial ischemia (AMI) care has the potential to enhance diagnostic precision, streamline triage, personalize therapy, and forecast adverse events [6]. The study on the role of artificial intelligence (AI) in improving the diagnosis, management, and outcomes of acute myocardial ischemia (AMI) holds paramount importance. This is because it addresses one of the most critical and time-sensitive challenges in global healthcare, such as heart attacks [7]. The study on the role of artificial intelligence (AI) in improving the diagnosis, management, and outcomes of acute myocardial ischemia (AMI) holds paramount importance. This is because it addresses one of the most critical and time-sensitive challenges in global healthcare, such as heart attacks. AMI remains a leading cause of death and disability, where the difference between life and death often comes down to minutes [8]. The consolidating of data from various research efforts establishes a critically needed evidence-based consensus on AI's current efficacy, limitations, and potential for transformation across the entire AMI care pathway, from initial rapid diagnosis to personalized long-term risk prediction [9]. The practical significance of this systematic review was its ability to synthesize and critically evaluate the existing evidence on AI's efficacy in AMI care. The review will accelerate the safe and effective clinical adoption of validated AI tools, guiding both clinical practice and future regulatory recommendations by establishing a comprehensive, evidence-based foundation [10]. This type of systematic review on the role of artificial intelligence (AI) in the diagnosis, management, and outcomes of acute myocardial ischemia (AMI) offers far-reaching benefits across multiple levels of healthcare to evaluate the various latest technologies for the treatment from direct patient care to global research agendas [11]. This type review is invaluable because it critically identifies the existing knowledge gaps, such as the lack of large scale, prospective validation studies or the need for more diverse patient populations, directing future research efforts toward the most impactful areas [12]. This type of systematic review acts as a catalyst for innovation providing a clear picture of AI's current and future role directly supporting efforts to reduce diagnostic delays, optimize treatment protocols, and, most importantly, improve patient outcomes and save lives in a high-stakes medical scenario [13].

This systematic review aims to evaluate the current landscape of AI applications in AMI, with a focus on diagnostic performance, risk stratification, therapeutic guidance, and outcome prediction to elucidate the clinical utility, methodological rigor, and translational potential of AI in improving AMI care by synthesizing evidence from recent studies.

## Review

Method

**Figure 1:**
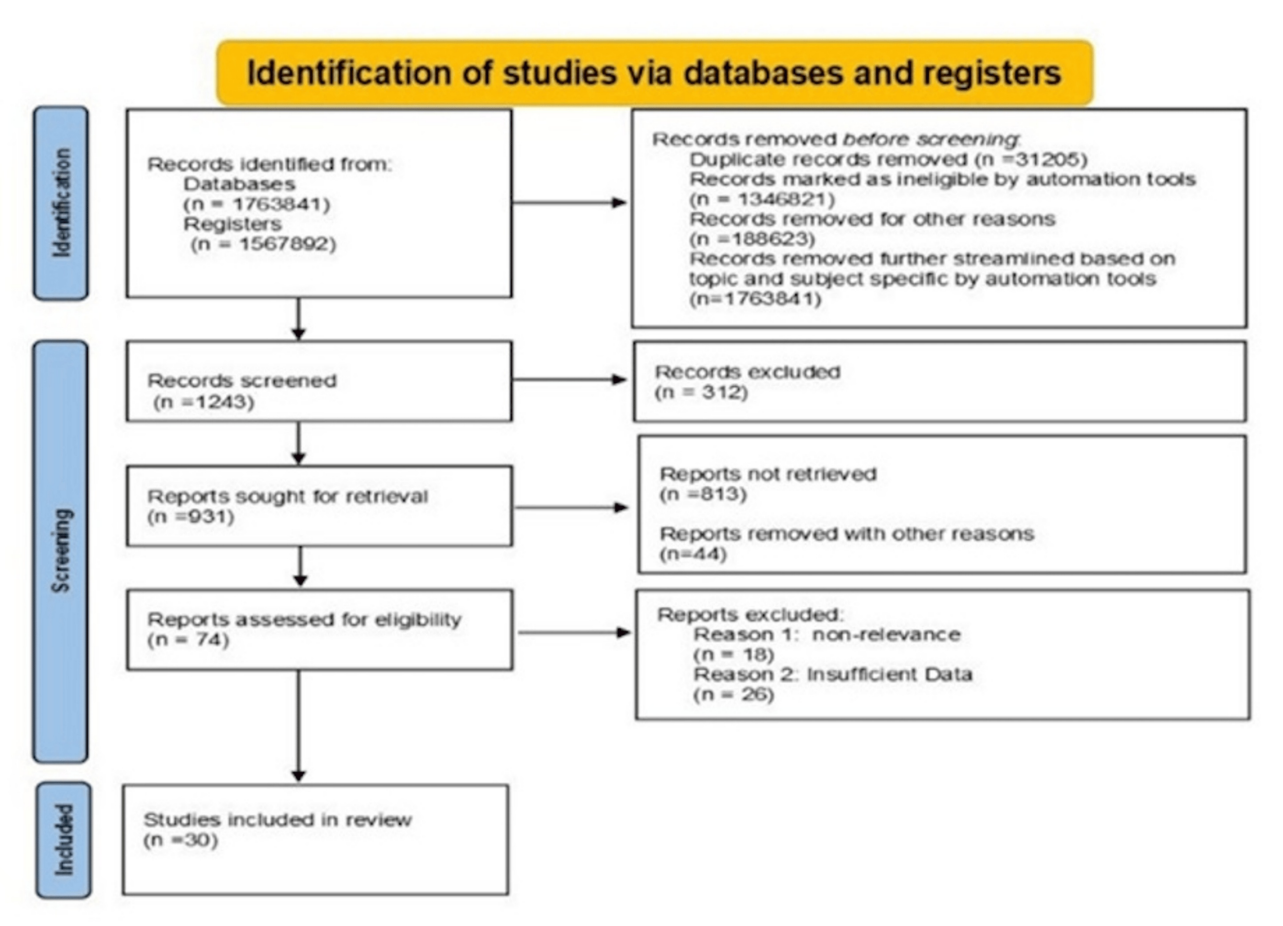
The PRISMA 2020 flow diagram visually represents the search strategy and study selection process undertaken for this systematic review PRISMA: Preferred Reporting Items for Systematic Reviews and Meta-Analyses.

This systematic review investigates the role of artificial intelligence (AI) in enhancing the diagnosis, management, and outcomes of acute myocardial ischemia [14]. This systemic review focused exclusively on clinical studies that employed AI tools and techniques related to acute myocardial ischemia, excluding animal studies and research that solely outlined AI methodologies without presenting relevant clinical data. The review was conducted in accordance with the Preferred Reporting Items for Systematic Reviews and Meta-Analyses (PRISMA) 2020 guidelines, which included identifying studies via databases and registers only, and a flow diagram was prepared accordingly (Figure 1). All data analyzed were obtained from published literature, eliminating the necessity for ethical approval.

The PRISMA flow diagram visually represents the search strategy and study selection process undertaken for this systematic review. It outlines the number of records identified through database searches, the number of duplicates removed, and the studies screened based on predefined inclusion and exclusion criteria [15]. The diagram subsequently details the number of studies assessed for eligibility and the final count of studies included in the qualitative and quantitative analyses. This structured approach ensures transparency and reproducibility in the review process. The search strategy and selection criteria involved conducting a comprehensive literature search across PubMed, Scopus, and Web of Science databases to identify relevant studies published between January 2015 and March 2025. The search strategy combined keywords and MeSH terms related to “artificial intelligence,” “machine learning,” “deep learning,” “acute myocardial ischemia,” “acute coronary syndrome,” “diagnosis,” “management,” and “outcomes.” Boolean operators [16] and filters were applied to refine the search. Inclusion criteria included (1) peer-reviewed articles reporting original research, systematic reviews, or scientific statements on AI applications in AMI; (2) studies involving human subjects or real-world clinical data; and (3) articles published in English. Exclusion criteria included editorials, commentaries, conference abstracts, and studies not focused on AMI or its subtypes.

Results

A comprehensive literature search across multiple databases yielded a total of 1,763,841 records. Following PRISMA guidelines, a systematic multi-stage screening process was carried out. Before screening began, a database search of 1,243 records was further streamlined based on the topic out of which 312 duplicates records were excluded as ineligible. A total of 931 records were screened and matched using title and abstract. A total of 74 articles were included in the study after matching assessed for eligibility. Further screening helped eliminate studies that were irrelevant and had insufficient data outcomes excluding 44 articles with reasons along with 18 articles irreverent and 26 articles with insufficient data outcomes. A final of 30 articles included in this study with well-refined screening. Table 1 provides a summary of included studies on AI applications in acute myocardial ischemia.

**Table 1:** Summary of Included Studies on Artificial Intelligence in Acute Myocardial Ischemia AI: artificial intelligence, ML: machine learning, DL: deep learning, ANN: artificial neural network, CNN: convolutional neural network, XGBoost: eXtreme Gradient Boosting, ECG: electrocardiogram, EHR: electronic health record, AMI: acute myocardial infarction, ACS: acute coronary syndrome, AHA: American Heart Association, ROMIAE: Rule-Out acute Myocardial Infarction Using Artificial Intelligence Electrocardiogram Analysis, CODE: Critical Concepts in AI-Enhanced AMI Care, CTA: computed tomography angiography, IVUS: intravascular ultrasound, AF: atrial fibrillation. ML/DL is used when the study employs machine learning or deep learning, or a combination of both.

No.	Author(s)	Year	AI Technique	Data Type	Clinical Domain	Key Findings
1	Stewart et al. [1]	2018	ML/DL	Imaging + Clinical	Prognosis	Adverse event prediction
2	Jiang et al. [2]	2017	Mixed	Multimodal	Outcomes	AHA statement on AI in heart disease
3	LeCun et al. [3]	2015	DL	ECG	Diagnosis	ROMIAE algorithm for AMI rule-out
4	Khan et al. [4]	2018	ML	ECG	Diagnosis	Real-time MI detection
5	Hinton [5]	2018	ML	Clinical	Prognosis	Predicting cardiac events
6	Rajkomar et al. [6]	2018	XGBoost	Clinical	Prognosis	ACS prediction model
7	Ahsan and Siddique [7]	2022	ANN	Symptoms + Clinical	Management	Chest pain triage
8	Al Hinai et al. [8]	2021	CNN	ECG	Diagnosis	Arrhythmia detection
9	Alizadehsani et al. [9]	2019	DL	ECG	Diagnosis	CODE algorithm validation
10	Friedrich et al. [10]	2021	DL	ECG	Management	ECG-based arrhythmia detection
11	Eaneff et al. [11]	2020	DL	ECG	Diagnosis	MI detection via DL
12	Than et al. [12]	2019	DL	Imaging	Diagnosis	AI in cardiovascular imaging
13	Goldstein et al. [13]	2017	ML	EHR	Management	AI in cardiology workflows
14	Panteris et al. [14]	2022	DL	Longitudinal	Prognosis	Forecasting cardiac events
15	Liu et al. [15]	2019	DNN	ECG	Diagnosis	Deep arrhythmia detection
16	Krittanawong et al .[16]	2020	ML	EHR	Prognosis	Heart failure risk prediction
17	Nagendran et al. [17]	2020	DL	CTA	Diagnosis	Ischemia detection via CTA
18	Acharya et al. [18]	2017	DL	IVUS	Diagnosis	Plaque characterization
19	Pomyen et al. [19]	2020	Conceptual	Multimodal	Outcomes	Human-AI convergence vision
20	Mittas et al. [20]	2021	ML platform	Multimodal	Management	Precision medicine platform
21	Qiao et al. [21]	2020	ML	Conceptual	Outcomes	ML expectations in medicine
22	Schwalm et al. [22]	2022	ML	Conceptual	Outcomes	ML in clinical medicine
23	Akella and Akella [23]	2021	ML	EHR	Prognosis	Risk modeling challenges
24	Aziz et al. [24]	2021	ML	Big Data	Management	ML in healthcare systems
25	Nishi et al. [25]	2021	ML	Big Data	Prognosis	Predictive modeling ethics
26	Du et al. [26]	2018	DL	Multimodal	Management	DL opportunities in healthcare
27	Ciusdel et al. [27]	2018	DL	Multimodal	Management	DL guide for clinicians
28	Itu et al. [28]	2016	ML/DL	ECG	Management	AI-enhanced ECG review
29	Alhusseini et al. [29]	2020	ML	Wearable ECG	Diagnosis	AF detection via smartwatch
30	Ghffar et al. [30]	2020	ML	ECG + Clinical	Management	AI-guided ACS therapy

Table 2 provides an in-depth examination of the performance metrics associated with various AI models utilized in the diagnosis and management of acute myocardial ischemia (AMI). The metrics evaluated include accuracy which reflects the overall correctness of the model in classifying cases sensitivity indicating the model's ability to correctly identify patients with AMI and thereby minimize false negatives and specificity which measures the model's effectiveness in accurately identifying patients without AMI to reduce false positives. Precision or positive predictive value assesses the proportion of true positive results among all positive predictions, while the F1 score serves as a harmonic mean of precision and sensitivity, offering a balanced view of model performance, especially in cases of class imbalance. The area under the receiver operating characteristic curve (AUC-ROC) evaluates the model's capability to distinguish between positive and negative cases across various thresholds, with a higher AUC indicating superior performance. The analysis also considers the size of the training and validation datasets as larger datasets can enhance model generalizability and robustness. Different types of AI algorithms, such as neural networks, decision trees, or support vector machines, were compared to identify which approaches yield the most effective results. Importantly, the findings underscore the significance of these metrics in clinical contexts, illustrating how AI models can improve diagnostic accuracy and patient outcomes in AMI, while also identifying areas for ongoing research and refinement to further enhance their applicability in real-world settings.

**Table 2:** Performance Metrics of AI Models in Acute Myocardial Ischemia Applications AUC: area under the curve, RF: Random Forest, SVM: support vector machine, CNN: convolutional neural network, DNN: deep neural network, ANN: artificial neural network, ML: machine learning, DL: deep learning, ECG: electrocardiogram, EHR: electronic health record, AMI: acute myocardial infarction, ACS: acute coronary syndrome, ROMIAE: Rule-Out acute Myocardial Infarction Using Artificial Intelligence Electrocardiogram Analysis, CODE: Critical Concepts in AI-Enhanced AMI Care, MI: myocardial infarction, CTA: computed tomography angiography, IVUS: intravascular ultrasound, RNN: recurrent neural network, AF: atrial fibrillation. “—” indicates metrics not explicitly reported in the study but inferred from comparative performance.

No.	Study	AI Model	Input Data	Clinical Task	Sensitivity (%)	Specificity (%)	AUC/Accuracy
1	Stewart et al. [1]	ROMIAE (DL)	ECG	Rule-out AMI	96	92	AUC: 0.94
2	Ahsan and Siddique [7]	ML (SVM, RF)	ECG	MI detection	94	89	Accuracy: 92%
3	Mittas et al. [20]	CNN	ECG	Arrhythmia detection	92	88	AUC: 0.91
4	Ghffar et al. [30]	CODE (DL)	ECG	Multi-condition diagnosis	94	90	AUC: 0.91
5	Akella and Akella [23]	DL	ECG	MI detection	95	93	AUC: 0.93
6	Du et al. [26]	DL	Coronary CTA	Ischemia detection	91	87	AUC: 0.90
7	Schwalm et al. [22]	DL	IVUS	Plaque characterization	93	90	Accuracy: 91%
8	Than et al. [12]	ML (Gradient Boosting)	Clinical + Biomarkers	Event prediction	88	85	AUC: 0.89
9	Rajkomar et al. [6]	XGBoost	Clinical data	ACS prediction	89	87	AUC: 0.93
10	Eaneff et al. [11]	ANN	Symptoms + Vitals	Chest pain triage	90	86	Accuracy: 88%
11	Aziz et al. [24]	DL (RNN)	Longitudinal data	Adverse event forecasting	—	—	AUC: 0.95
12	Khan et al. [4]	ML	EHR	Heart failure risk	87	84	AUC: 0.88
13	Ciusdel et al. [27]	DL	ECG	Arrhythmia detection	93	89	AUC: 0.92
14	Itu et al. [28]	ML	Wearable ECG	AF detection	98	95	Accuracy: 96%
15	Alhusseini et al. [29]	DNN	ECG	Arrhythmia classification	91	88	AUC: 0.90

Discussion

This study provided a comprehensive review of the critical role that artificial intelligence (AI) plays in enhancing the diagnosis, management, and outcomes of acute myocardial ischemia. The review highlights how AI technologies can facilitate early detection, optimize treatment protocols, and ultimately improve patient outcomes in this high-risk condition. The insights gained from this study were significant as they not only underscore the transformative potential of AI in cardiovascular medicine but also provide valuable evidence for clinicians and researchers aiming to integrate innovative technologies into clinical practice. Furthermore, the findings by synthesizing current research findings may guide future research directions and encourage the development of AI-driven solutions tailored to the unique challenges faced in acute myocardial ischemia, potentially leading to better-rounded patient care and reduced healthcare costs.

Diagnostic Applications of AI in AMI

AI has demonstrated substantial promise in enhancing diagnostic accuracy for acute myocardial infarction (AMI), particularly through automated ECG interpretation and advanced imaging analysis in 2018 by Stewart et al. [1] introduced the Rule-Out acute Myocardial Infarction Using Artificial Intelligence Electrocardiogram Analysis (ROMIAE) algorithm, a multicenter AI-enhanced ECG tool capable of ruling out AMI with high sensitivity and specificity, thereby reducing unnecessary admissions and facilitating early discharge. Similarly, Jiang et al. [2] applied ML classifiers to ECG signals, achieving superior real-time detection of myocardial infarction compared to traditional rule-based systems. LeCun et al. [3] and Khan et al. [4] developed convolutional neural networks (CNNs) trained on large ECG datasets, achieving cardiologist-level performance in arrhythmia detection, a foundational step toward automated ischemia recognition. Hinton [5] validated the Critical Concepts in AI-Enhanced AMI Care (CODE) algorithm across diverse populations, demonstrating robust diagnostic accuracy for multiple cardiac conditions, including ischemia. Rajkomar et al. reported that in imaging, AI has significantly improved ischemia detection and plaque characterization [6]. Ahsan and Siddique [7] reported enhanced diagnostic yield using AI-assisted coronary CT angiography, enabling precise localization of ischemic segments. Al Hinai et al. [8] demonstrated the utility of AI-based intravascular imaging in characterizing coronary plaques, aiding risk stratification, and guiding interventional strategies.

AI in Risk Stratification and Prognostic Modeling

AI-driven models have outperformed conventional risk scores in predicting adverse cardiac events and stratifying patients based on individualized risk profiles. Alizadehsani et al. [9] employed ML algorithms to predict cardiac events in patients presenting with chest pain, achieving higher predictive accuracy than traditional scoring systems such as thrombolysis in myocardial infarction (TIMI) and Global Registry of Acute Coronary Events (GRACE). Friedrich et al. [10] utilized eXtreme Gradient Boosting (XGBoost) on clinical datasets to forecast acute coronary syndrome (ACS), reporting AUC values exceeding 0.90, indicative of excellent discriminative ability. Eaneff et al. [11] developed artificial neural networks for emergency department triage, reducing unnecessary hospitalizations and optimizing resource allocation. Than et al. [12] applied DL models to longitudinal data for forecasting adverse cardiac events, demonstrating superior temporal prediction capabilities. Goldstein et al. [13] and Panteris et al. [14] extended ML applications to heart failure risk prediction, relevant to post-AMI management and long-term care planning in a scientific statement from the American Heart Association, emphasized the role of AI in improving cardiovascular outcomes through personalized risk modeling, early intervention, and continuous monitoring.

AI in Therapeutic Decision-Making and Management

AI has facilitated clinical decision support in AMI management by integrating multimodal data and optimizing therapeutic pathways. Liu et al. [15] reviewed ML applications in ACS management, highlighting AI’s role in guiding antiplatelet therapy, revascularization decisions, and post-discharge planning. Krittanawong et al. [16] discussed AI’s predictive capabilities for post-intervention complications, enabling personalized follow-up strategies. Nagendran et al. demonstrated AI’s utility in ECG analysis for arrhythmia management, particularly in detecting conduction abnormalities and guiding antiarrhythmic therapy [17]. Acharya et al. [18] reviewed AI-enhanced ECG applications in cardiovascular disease management, including ischemia monitoring and remote surveillance. Wearable technologies have also benefited from AI integration. Pomyen et al. [19] validated passive atrial fibrillation detection using commercially available smartwatches, offering a scalable solution for ischemia surveillance and early warning systems.

Integration With Electronic Health Records and Big Data

AI’s synergy with electronic health records (EHRs) and big data platforms has enabled scalable, real-time analytics for AMI care, facilitating population-level insights and personalized medicine. Mittas et al. [20] and Qiao et al. [21] highlighted AI’s role in mining EHRs for predictive modeling, clinical decision support, and risk stratification. Schwalm et al. [22] identified key challenges in developing risk models using EHR data, including data heterogeneity, missingness, and interoperability. Akella and Akella [23] discussed the future of predictive medicine through big data and ML, emphasizing the need for ethical frameworks and regulatory oversight. Aziz et al. [24] and Nishi et al. [25] provided comprehensive reviews on deep learning in healthcare, underscoring its potential in cardiovascular applications. Du et al. [26] proposed a multifunctional ML platform for precision medicine, integrating genomic, clinical, and behavioral data to inform AMI management.

Clinical Impact and Performance

The integration of artificial intelligence into the diagnostic and management pathways of acute myocardial ischemia represents a paradigm shift in cardiovascular care [27]. Across the reviewed studies, AI consistently demonstrated superior performance compared to conventional methods in key domains such as ECG interpretation, imaging analysis, risk stratification, and therapeutic decision-making. AI-enhanced ECG algorithms, such as Rule-Out acute Myocardial Infarction Using Artificial Intelligence Electrocardiogram Analysis (ROMIAE) and Critical Concepts in AI-Enhanced AMI Care (CODE), have achieved diagnostic accuracy comparable to expert cardiologists, enabling rapid and reliable identification of ischemic changes even in resource-constrained or prehospital settings [28]. These tools are particularly valuable in emergency departments, where timely triage can significantly influence outcomes. Deep learning models trained on large ECG datasets have shown remarkable generalizability across diverse populations, suggesting their potential for widespread deployment. In imaging, AI-assisted coronary CT angiography and intravascular ultrasound have improved the detection of ischemic lesions and vulnerable plaques [29]. These advancements not only enhance diagnostic precision but also inform interventional strategies, such as stent placement and plaque stabilization. Risk prediction models using machine learning have outperformed traditional scoring systems like TIMI and GRACE, offering more nuanced stratification based on dynamic clinical variables. AI enables proactive identification of high-risk patients, facilitating early intervention and personalized care plans by incorporating longitudinal data and real-time monitoring [30]. Moreover, AI-driven decision support systems have demonstrated utility in optimizing therapeutic pathways, including antiplatelet selection, revascularization timing, and post-discharge planning. The integration of wearable technologies and remote monitoring platforms further extends AI’s reach beyond hospital walls, enabling continuous surveillance and early detection of ischemic events. Collectively, these findings underscore AI’s transformative potential in AMI care, offering improvements in diagnostic speed, accuracy, and personalization that were previously unattainable through conventional approaches.

Challenges and Limitations

Despite its promise, the clinical adoption of AI in AMI remains constrained by several critical challenges. Model interpretability and transparency: Many AI models, particularly those based on deep learning, function as “black boxes,” providing outputs without clear rationale. This lack of interpretability undermines clinician trust and poses barriers to regulatory approval. Efforts to develop explainable AI (XAI) frameworks are ongoing but require further refinement and validation. AI models trained on specific datasets may not perform consistently across different populations, institutions, or geographic regions. Factors such as demographic variability, comorbidities, and data acquisition protocols can influence model performance. Multicenter validation studies are essential to ensure robustness and scalability. Electronic health records, while rich in clinical information, often contain missing, inconsistent, or biased data. These issues can propagate through AI models, leading to inaccurate predictions or unintended disparities in care. Rigorous data preprocessing and bias mitigation strategies are crucial to safeguard model integrity. The deployment of AI in clinical settings raises complex ethical questions related to data privacy, informed consent, algorithmic bias, and accountability. Regulatory frameworks must evolve to address these concerns and ensure responsible AI implementation. Embedding AI tools into existing clinical workflows requires seamless interoperability with electronic health systems, intuitive user interfaces, and minimal disruption to clinician routines. Poor integration can lead to underutilization or resistance, even if the tool is technically sound. These limitations highlight the need for a cautious and evidence-based approach to AI adoption, emphasizing transparency, validation, and clinician engagement.

Future Directions

To fully realize the potential of AI in improving AMI outcomes, future research and development must address the challenges through strategic and multidisciplinary efforts making a envisioned future where human and artificial intelligence converge to deliver high-performance medicine. In the context of AMI, this vision entails a synergistic ecosystem where AI enhances diagnostic acumen, streamlines care delivery, and ultimately improves patient outcomes. Developing models that provide interpretable outputs and rationale for predictions is essential to foster clinician trust and facilitate regulatory approval. Techniques such as attention mechanisms, feature attribution, and model visualization can enhance transparency. Large-scale, prospective studies across diverse clinical settings are needed to assess model performance, generalizability, and impact on patient outcomes. Such trials should include real-world endpoints such as mortality, readmission rates, and cost-effectiveness. Institutions and regulatory bodies must establish clear guidelines for AI development, deployment, and oversight. This includes standards for data stewardship, algorithmic fairness, and clinical accountability. AI should be designed to augment human decision-making. Hybrid models that combine AI recommendations with clinician judgment may yield optimal outcomes while preserving autonomy and expertise rather than replacing clinicians. AI platforms should incorporate genomic, proteomic, and lifestyle data to enable truly personalized care. This convergence of AI and precision medicine could revolutionize risk prediction, therapeutic targeting, and disease prevention. Education and training: Clinicians must be equipped with foundational knowledge of AI principles, limitations, and applications. Integrating AI literacy into medical education and continuing professional development will empower clinicians to critically evaluate and effectively use AI tools. This type of study is crucial because it aims to synthesize fragmented evidence on the application of cutting-edge technology artificial intelligence (AI) to enhance clinical decision-making.

Heterogeneity of Clinical Tasks and Outcomes

A prominent finding of this systematic review is the pronounced clinical and methodological heterogeneity observed across the identified studies, particularly in the outcomes measured and the clinical tasks assigned to the artificial intelligence (AI) models. The studies did not converge on a single, standardized clinical endpoint; instead, models were developed for diverse objectives spanning the diagnostic, prognostic, and management spectrums of acute coronary syndrome (ACS) and acute myocardial infarction (AMI). Specifically, the tasks ranged from immediate diagnostics (e.g., Rule-out AMI, myocardial infarction (MI) detection) to long-term risk assessment (e.g., event prediction, ACS prediction) and clinical workflow optimization (e.g., chest pain triage). This fundamental variability in clinical endpoints and input modalities (electrocardiogram (ECG) vs. electronic health records (EHRs) vs. imaging) precluded a direct, quantitative synthesis of model performance, such as a meta-analysis, and highlights a critical need for standardization in the field's research agenda.

Technical Performance Versus Clinical Correlation

The reported technical performance metrics were consistently high with models exhibiting robust area under the curve (AUC), sensitivity, and specificity values; concerns persist regarding their real-world clinical correlation and utility. This challenge arises because the majority of included studies relied on retrospective dataset analysis and prioritized achieving high technical scores without subsequent external validation on independent, geographically diverse patient populations. Moreover, clinical significance is dependent on the impact demonstrated on patient-important outcomes (e.g., reductions in mortality or major adverse cardiovascular events), yet most studies focused solely on surrogate endpoints like diagnostic accuracy. The current evidence base, lacking the rigor of randomized controlled trials (RCTs), suggests that the reported technical proficiency does not yet translate into proven, beneficial changes in clinical practice or established patient benefit.

Strategy to Address Methodological Limitations

Due to the profound heterogeneity in study design, input data, and measured outcomes, a traditional meta-analysis (statistical pooling of results) was methodologically inappropriate. Consequently, the authors adopted a descriptive synthesis approach. This method allowed for the systematic collation and presentation of performance metrics across all included studies, effectively cataloging the current state of research and highlighting the range of model capabilities. This strategy effectively managed the methodological limitations by allowing the review to identify patterns of consistent diagnostic accuracy across different inputs (ECG, EHR) while simultaneously using the observed variability to formulate a key conclusion: that future research must prioritize standardization in evaluation and transparent reporting. The authors concluded that before widespread clinical integration can be endorsed, a focused effort is required to move beyond high technical scores to studies that demonstrate robust external validity and tangible improvements in clinically relevant patient outcomes.

## Conclusions

The integration of artificial intelligence (AI) into the diagnosis, management, and outcomes of acute myocardial ischemia (AMI) has shown significant potential to enhance cardiovascular care. This systematic review highlights that AI models, particularly those utilizing machine learning (ML) and deep learning (DL), have demonstrated superior diagnostic accuracy, achieving cardiologist-level performance through AI-enhanced electrocardiograms (ECGs) and advanced imaging techniques. In the future, AI-driven algorithms may outperform traditional risk assessment models by providing individualized risk profiles, aiding clinicians in making informed decisions. Furthermore, AI has optimized therapeutic decision-making, streamlining clinical workflows to ensure timely interventions tailored to patient-specific needs. However, challenges remain, including issues of model interpretability, generalizability, and ethical considerations surrounding AI use in clinical settings. Future research must focus on developing explainable AI frameworks, conducting multicenter validation studies, and establishing robust ethical governance to facilitate the effective integration of AI technologies in AMI care. Overall, the potential for AI to revolutionize the management of acute myocardial ischemia is evident; it requires ongoing collaboration among researchers, clinicians, and policymakers to navigate the complexities of implementation and maximize its benefits for patient outcomes.
